# Catastrophizing and pain impact in migraineurs

**DOI:** 10.1186/1129-2377-14-S1-P147

**Published:** 2013-02-21

**Authors:** C Pires, E Solé, J Miró

**Affiliations:** 1 Universitat Rovira i Virgili, Spain

## Introduction

One of the important determinants of the chronic pain experience is pain catastrophizing[1,2]. In migraine, a handful of works have studied the relationship between catastrophizing and pain, i.e. its role in the development and/or maintenance of migraine episodes, and its interactions with other psychological variables (coping strategies, anxiety, depression and personality traits). The aims of this study were (1) to examine whether there are differences among migraineurs and nonmigraineurs in catastrophizing, as well as on the psychological variables, (2) to explore the relationship between these variables and catastrophizing in the migraine group, and (3) to put into relation the headache impact with the other studied variables. METHOD:81 subjects participated in this study (43 migraineurs). 81.5% were women, mean age was 30.2 (SD=10.93) and 67% were students. Personality, depression, anxiety, coping strategies, catastrophizing and headache pain impact were measured.

## Results

Catastrophizing was positively related to depression (r=0.40, p<.01), anxiety (r=0.44, p<.01), and aggression-hostility (r=0.39, p<.05), and negatively to distraction (r= -0.37, p<.05). Regression analyses revealed anxiety as a unique predictor of catastrophizing (R2=0.19, p<.05) and headache impact (R2= 0.32, p<.01). Migraineurs reported higher scores on catastrophizing, anxiety, and personality dimensions: activity, neuroticism-anxiety and aggression-hostility compared to nonmigraineurs. Nonmigraineurs used more passive coping strategies, specifically catharsis. Migraine characteristics (pain intensity, frequency and duration) were not associated with catastrophizing.

## Conclusions

Results show that migraineurs present distinctive psychological characteristics from nonmigraineurs. Catastrophizing was one of them, which was predicted by anxiety that also predicted the impact of headaches. Additional research is needed to explore the link between anxiety, catastrophizing and migraine. The role of these psychological factors should be specially considered when treating migraine.
